# Supplementary material for: Reproducible Bioinformatics Analysis Workflows for Detecting IGH Gene Fusions in B-Cell Acute Lymphoblastic Leukaemia Patients
Source: Cancers (Basel). 2023 Sep 26;15(19):4731. doi: 10.3390/cancers15194731 (PMC10571859; doi:10.3390/cancers15194731)
Supplement: Supplementary file 1 [file cancers-15-04731-s001.zip › Analysis-Workflow_B-ALL_SupplementaryData.pdf]

Supplementary Data file

Supplementary Data S1

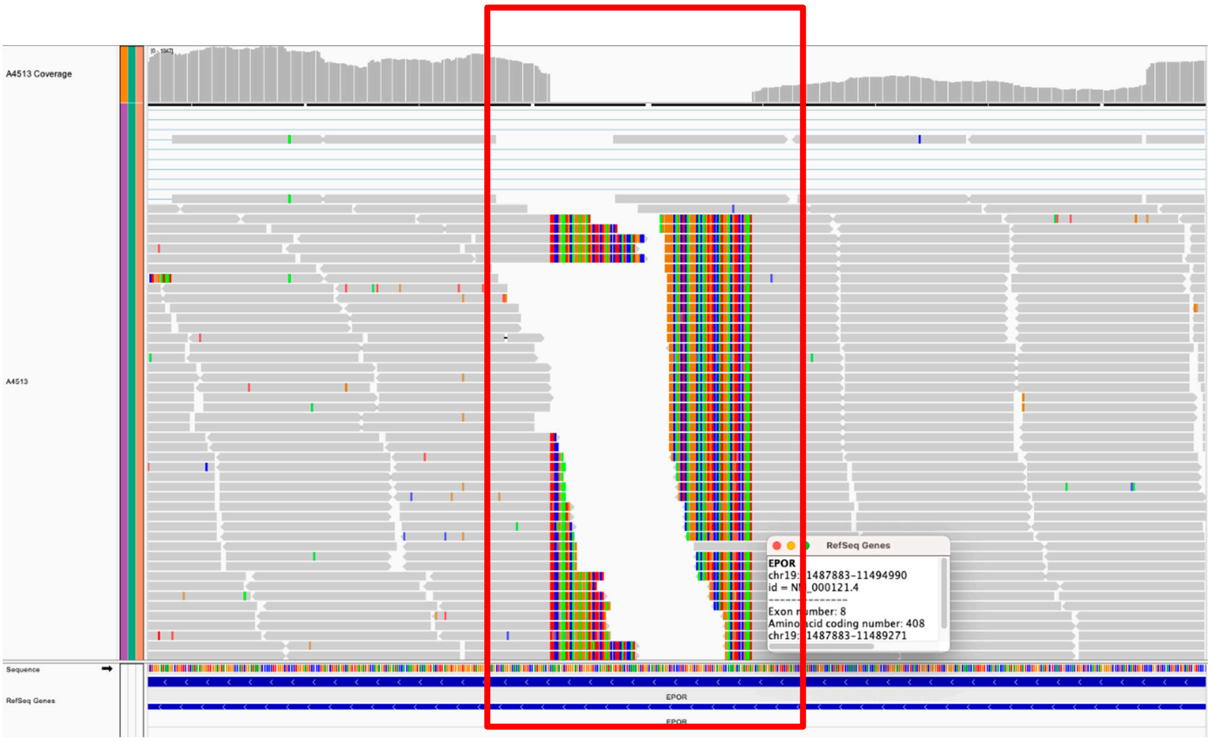

Identification of *IGH::EPOR* gene fusions. *IGH::EPOR* fusions were confirmed through inspection of the sequencing reads in the BAM using IGV (Integrative Genomics Viewer). Above you can see the mismatches at the end of the sequencing reads that are mapped to *EPOR*.

Supplementary Data S2
The default commands used to run the standard pipeline.

Gene Fusion Detection Algorithm	Version	Alignment algorithm (version)	Default parameters
Arriba	2.1.0	STAR (v2.7.9a)	`arriba \`<br>`-x /dev/stdin \`<br>`-o ${filename}.fusions.tsv \`<br>`-O`<br>`${filename}.fusions.discarded.`<br>`tsv \`<br>`-a ${assembly} \`<br>`-g ${gtf} \`<br>`-b ${blacklist} \`<br>`-k ${knownfus} \`<br>`-p ${proteindom}`
FusionCatcher	1.33	Bowtie (v1.2.3)<br>Bowtie2 (v2.3.5.1)<br>STAR (v2.7.2b)	`fusioncatcher \`<br>`-p ${task.cpus} \`<br>`-d ${fusioncatcher_db} \`<br>`-i ${reads[0]},${reads[1]} \`<br>`-o \${PWD} \`<br>`--skip-blat`
Star-Fusion	1.10.0	STAR (v2.7.8)	`STAR-Fusion \`<br>`--genome_lib_dir ${ctat_dir} \`<br>`--left_fq ${reads[0]} \`<br>`--right_fq ${reads[1]} \`<br>`--CPU ${task.cpus} \`<br>`--output_dir \${PWD}`

Supplementary Data S3

The default and altered values for the parameters used for each test of STAR-Fusion. Results from Read Support (3) and Read Support + FFPM (3) were used for the manuscript as they were the best performing for the given parameter combination.

Test Name	Parameter	Default Value	Altered Value
**Read Support (1)**	`--min_novel_junction_support`	1	0
**Read Support (2)**	`--min_junction_reads`	2	1
**Read Support (3)**	`--min_junction_reads`<br>`--min_novel_junction_support`	1<br>2	0<br>1
**Read Support + FFPM (1)**	`--min_novel_junction_support`<br>`--min_FFPM`	1<br>0.1	0<br>0.001
**Read Support + FFPM (2)**	`--min_junction_reads`<br>`--min_FFPM`	2<br>0.1	1<br>0.001
**Read Support + FFPM (3)**	`--min_junction_reads`<br>`--min_novel_junction_support`<br>`--min_FFPM`	1<br>2<br>0.1	0<br>1<br>0.001
**Max Sensitivity**	`--max_sensitivity`<br>Includes:<br>`--min_junction_reads`<br>`--min_sum_frags`<br>`--require_LDAS`<br>`--min_spanning_frags_only`<br>`--min_novel_junction_support`<br>`--skip_FFPM`<br>`--`<br>`no_single_fusion_per_breakpoint`<br>`--skip_EM`	 <br>1<br>2<br>1<br>5<br>3<br>0.1<br>n/a<br>n/a	 <br>0<br>1<br>0<br>1<br>1<br>n/a<br>TRUE<br>TRUE
**Full Monty**	`--full_Monty`<br>Includes `--max_sensitivity`, and:<br>`--max_promiscuity`<br>`--min_pct_dom_promiscuity`<br>`--min_alt_pct_junction`<br>`--no_annotation_filter`<br>`--no_RT_artifact_filter`	 <br>10<br>20<br>10.0<br>n/a<br>n/a	 <br>1000000<br>1<br>1e-3<br>TRUE<br>TRUE
